# Functional Analysis of 3′UTR Variants at the *LDLR* and *PCSK9* Genes in Patients with Familial Hypercholesterolemia

**DOI:** 10.1155/2024/9964734

**Published:** 2024-02-08

**Authors:** Javier Sanguino Otero, Carmen Rodríguez-Jiménez, Jose Mostaza Prieto, Carlos Rodríguez-Antolín, Ana Carazo Alvarez, Francisco Arrieta Blanco, Sonia Rodríguez-Nóvoa

**Affiliations:** ^1^Department of Genetics of Metabolic Diseases, Hospital Universitario La Paz, Madrid, Spain; ^2^Dyslipidemia of Genetic Origin and Metabolic Diseases Group, IdiPAZ, Hospital Universitario La Paz, Madrid, Spain; ^3^Lipid and Vascular Unit, Hospital Carlos III, Madrid, Spain; ^4^Cancer Epigenetics Laboratory, INGEMM, La Paz University Hospital, Madrid, Spain; ^5^Biomarkers and Experimental Therapeutics in Cancer, IdiPAZ, Madrid, Spain; ^6^Department of Endocrinology and Nutrition, Hospital Ramón y Cajal, Madrid, Spain; ^7^Instituto Ramón y Cajal de Investigación Sanitaria (IRyCIS), E-28034 Madrid, Spain; ^8^CIBER de Fisiopatología de la Obesidad y Nutrición (CIBEROBN), Madrid, Spain

## Abstract

Familial hypercholesterolemia (FH) is an autosomal dominant disease with an estimated prevalence of 1 in 200-250 individuals. Patients with FH are at increased risk of premature coronary artery disease. Early diagnosis and treatment are essential for improving clinical outcomes. In many cases, however, the genetic diagnosis is not confirmed. At present, routine genetic testing does not analyze the 3′UTR regions of *LDLR* and *PCSK9*. However, 3′UTR-single nucleotide variants could be of interest because they can modify the target sequence of miRNAs that regulate the expression of these genes. Our study fully characterizes the 3′UTR regions of *LDLR* and *PCSK9* in 409 patients with a suspected diagnosis of FH using next-generation sequencing. In 30 of the 409 patients, we found 21 variants with an allelic frequency of <1%; 14 of them at 3′UTR-*LDLR* and 8 at 3′UTR-*PCSK9*. The variants' pathogenicity was studied *in silico*; subsequently, a number of the variants were functionally validated using luciferase reporter assays. *LDLR*:c.^∗^653G > C showed a 41% decrease in luciferase expression, while *PCSK9*:c.^∗^950C > T showed a 41% increase in PCSK9 expression, results that could explain the hypercholesterolemia phenotype. In summary, the genetic analysis of the 3′UTR regions of *LDLR* and *PCSK9* could improve the genetic diagnosis of FH.

## 1. Introduction

Familial hypercholesterolemia (FH) is a genetic dyslipidemia with an estimated prevalence of 1 in 200-250 inhabitants for the heterozygous form (HeFH) [1] and 1-9 in 1,000,000 inhabitants for the homozygous form [2]. Patients with FH typically have elevated serum cholesterol levels and an increased risk of premature coronary heart disease [3, 4]. Pathogenic variants in the low-density lipoprotein receptor (*LDLR*), apolipoprotein B (*APOB*), and proprotein convertase subtilisin/kexin type 9 (*PCSK9*) genes cause HeFH, with over 1800 mutations reported [5–8]. There are also very rare recessive forms of hypercholesterolemia caused by biallelic pathogenic variants at *LDLRAP1* [9].

The diagnosis of FH is based on clinical and biochemical findings. The Dutch Lipid Clinic Network Score criterion is widely used in clinical practice and categorizes FH into definite, probable, possible, or unlikely [10]. However, genetic confirmation is advisable because carriers of pathogenic variants show a higher risk of cardiovascular disease (CVD) than noncarriers [4]. The detection of a causative genetic variant confirms the diagnosis of FH and enables the testing of family members for the early detection of new cases of FH.

Next-generation sequencing is currently employed for genetic testing and has improved the diagnostic yield significantly compared with previous technologies; however, there are still numerous patients with a clinical diagnosis of FH without genetic confirmation [11–14]. The lack of genetic confirmation in most cases could be due to undescribed genes that could be associated with FH; however, the study of the complete exome found no new candidate genes [15]. The FH phenotype has a certain overlap with polygenic hypercholesterolemia, which could partially explain the lack of concordance between genetic and clinical diagnoses.

The routine sequencing analysis of FH does not include deep intronic or untranslated regions (UTRs) of *LDLR*, *APOB*, or *PCSK9* despite the fact that several pathogenic variants have been reported in these regions [16–18]. A number of studies on 3′UTR-*LDLR* and *PCSK9* regions have suggested that 3′UTR variants could affect these genes' expression by disrupting microRNA-mRNA binding and causing hypercholesterolemia [19, 20].

miRNAs are short (approximately 22 nt), endogenous, noncoding, single-stranded RNAs that act as important elements in the posttranscriptional regulation of gene expression [21, 22]. miRNAs recognize their target mRNAs through imperfect pairing with the 3′UTR of mRNAs [23]. In recent years, several studies have demonstrated that certain miRNAs (such as miR148a, miR128-1, miR185, miR-34a, and miR27a) could play a pivotal role in LDL-c metabolism [24–27]. These miRNAs regulate the expression of genes such as *LDLR*, *PCSK9*, and *ABCA1* through targets in the 3′UTR region of these genes. In vivo studies have shown that genetically modified mice underwent overexpression or inhibition of these miRNAs, causing significant changes in plasma LDL-c levels [25, 28].

A number of studies have reported an association between common polymorphisms in the *LDLR*-3′UTR and *PCSK9*-3′UTR regions with LDL-c levels [19, 20, 29], low HDL-c levels [30], and the response to lipid-lowering drugs [31, 32]. Dysregulation of miRNA binding by 3′UTR polymorphisms has been proposed as the underlying mechanism of lipid abnormalities.

Our hypothesis is that rare variants in 3′UTR in *LDLR* and *PCSK9* could have a significant effect on the expression of these genes due to miRNA dysregulation. The objective of this study was to evaluate the impact and pathogenicity of rare 3′UTR variants in *LDLR* and *PCSK9* found in a large patient group with suspected FH. To this end, we analyzed the 3′UTR region, selected variants using *in silico* tools, and then functionally characterized these variants using luciferase reporter assays.

## 2. Patients and Methods

The study was conducted in four stages:

1. Characterization of the study population
2. Analysis of the 3′UTR region of the *LDLR* and *PCSK9* genes
3. *In silico* analysis of 3′UTR-*LDLR* and *PCSK9* variants
4. Functional studies to evaluate the impact of selected variants at 3′UTR-*LDLR* and *PCSK9*

### 2.1. Study Participants

The study analyzed a group of 409 unrelated patients with suspected FH in the Genetic Metabolic Disorders Laboratory at La Paz University Hospital, Madrid (Spain). All patients were referred to our center for routine genetic analysis of hypercholesterolemia and had a clinical history of high LDL-cholesterol (LDL-c) levels, a family history of CVD, a family history of dyslipidemia, and/or a personal history of early CVD. The study protocol was approved by the local ethics committee, and all participants signed the written informed consent document.

### 2.2. Genetic Analysis

Extraction of genomic DNA from whole EDTA blood was performed using the Chemagen kit (Chemagic DNA extraction special, PerkinElmer Inc, Baesweiler, Germany), and DNA concentrations were measured in a NanoDrop ND-1000 Spectrophotometer.

Genetic analysis was performed by next-generation sequencing (NGS) of *LDLR*, *APOB*, *PCSK9*, and *LDLRAP1* using a customized panel (see Supplementary Table SPTB1). The panel was especially designed to cover the complete 3′UTR region of *LDLR* and *PCSK9*. The DNA library was prepared, and the exome enrichment steps were conducted according to the supplier's recommendations (NimbleGen, Roche), and sequencing was performed on a MiSeq or NextSeq system sequencing (Illumina). The quality parameter of more than 30× reads in 99% of the targets was considered before the NGS data analysis. Genetic variants of interest were confirmed by the Sanger sequencing. Copy number variants were analyzed *in silico* and were subsequently confirmed by multiplex ligation-dependent probe amplification (MLPA, Salsa P062-D2 kit, MRC-Holland, Amsterdam).

The bioinformatics analysis was performed using customized algorithms. Briefly, sequences were mapped to the CRCh37/hg19 human reference sequence. The following predictors of pathogenicity were employed: combined annotation-dependent depletion, sorting tolerant form intolerant, polymorphism phenotyping v2, mutation assessor, functional analysis through hidden Markov models, and the variant effect scoring tool. The splicing predictors employed were NNSplice, GeneSplicer, and Human Splicing Finder. Variants were classified according to the American College of Medical Genetics and Genomics guidelines [33].

### 2.3. *In Silico* Analysis of 3′UTR Variants

The effect of the 3′UTR variants found at *LDLR* and *PCSK9* with an allelic frequency < 0.01 was analyzed with the miRanda algorithm (http://www.microrna.org/) [34]. This bioinformatics tool compares the alignment between sequences with 3′UTR variants or WT sequence and miRNAs reported in the miRBase database (http://www.mirbase.org/) [35]. The results are categorized into the following three types:

1. miRNAs added: new illegitimate miRNA binding sites created by the 3′UTR variants that do not carry the WT sequence
2. miRNAs removed: binding miRNA sites in the WT sequence removed in the sequence with 3′UTR variants
3. miRNAs modified: binding miRNA sites in the WT sequence modified in the sequence with 3′UTR variants, making a weaker binding

The miRNAs employed in the functional validation of the variants were selected based on the miRanda prediction and then validated with the following 3 bioinformatics tools: miRWalk3.0 (http://mirwalk.umm.uni-heidelberg.de/), TargetScan (http://www.targetscan.org/), and miRDB (http://www.mirdb.org/) [36–38].

### 2.4. Functional Characterization of *LDLR* and *PCSK9*-3′UTR Variants

#### 2.4.1. Cell Culture of HepG2

The HepG2 cell line was cultured in Dulbecco's Modified Eagle Medium (Gibco, Life Technologies, Carlsbad, CA, USA) supplemented with 10% heat-inactivated fetal bovine serum (Gibco, Life Technologies, Carlsbad, CA, USA), 100 U/mL penicillin, 100 *μ*g/mL streptomycin, and 2 mmol/L L-glutamine. HepG2 cells were grown in a monolayer culture in 75 cm^2^ flasks and incubated at 37°C in a 5% CO_2_ atmosphere.

#### 2.4.2. Site-Directed Mutagenesis

The *LDLR*-3′*UTR* construct (*LDLR* NM_00052 human 3′UTR clone, WT) and the *PCSK9*-3′ UTR construct (*PCSK9* NM_174936 human 3'UTR clone, WT) were obtained from OriGene to perform the luciferase reporter experiment assays. The complete sequence of *LDLR*-3′ UTR wild type (WT) or *PCSK9*-3′ UTR WT was added downstream of the firefly luciferase gene in the 3′UTR WT constructs. miRNA can selectively bind to the added 3′UTR sequence and decrease the firefly luciferase expression. These constructs were expanded in *E. coli* for subsequent transfection experiments. The 3′UTR constructs were obtained using the QuikChange lightning site-directed mutagenesis kit (Agilent Technologies) according to the manufacturer's instructions and subsequently expanded in *E. coli*. Mutated-plasmid sequences were validated by the Sanger sequencing.

#### 2.4.3. Transfection and Luciferase Reporter Assay

The reporter luciferase assay was used for the experiments, comparing the luciferase expression of constructs *LDLR*-3′UTR and *PCSK9*-3′UTR with WT 3′UTR to assess the effect of the different variants. The dual luciferase reporter assay system (Promega) was employed to measure the luciferase activity of the HepG2 lysates in a luminometer (Tecan 200 Infinity). All luciferase reporter luminescence measurements were normalized with Renilla luminescence cotransfected in all the samples. Experiments were performed in triplicate and in three independent experiments. Luciferase expression was determined as the mean of the three measurements.

#### 2.4.4. Cotransfection of 3′UTR Constructs with miRNA Mimics or AntimiRNA Inhibitor

To assess the effect of the 3′UTR variants on miRNA binding, we performed experiments on the overexpression and inhibition of miRNAs using miRNA mimics and antimiRNA inhibitors. Inhibition assays with inhibitors help confirm the direct binding of the miRNA to the target gene. The decrease in luciferase expression caused by an miRNA that binds its target gene must then be reversed by the inhibitors. The WT 3′ UTR construct or mutated-plasmid construct was cotransfected with miRNA mimics or mimic negative control (NC, Invitrogen™ Thermo Fisher Scientific, Waltham, USA). These experiments help probe the creation of illegitimate miRNA binding sites in *LDLR* or the removal of miRNA binding sites in *PCSK9*.

HepG2 cells were seeded in 24-well plates at 18 × 10^4^/well, incubated at 37°C under a 5% CO_2_ atmosphere for 48 h to reach a confluence of approximately 50%–70%, and then transiently transfected. We cotransfected 2.5 *μ*g of each of the 3′UTR constructs, and 0.15 mM of miRNA mimic or miRNA mimic negative control (mimic NC) was cotransfected using Lipofectamine 3000 and Opti-MEM serum-free medium according to the manufacturer's instructions (Invitrogen™).

Renilla luciferase reporter was also cotransfected in all samples to normalize the luciferase reporter signal. An empty vector was employed as a transfection control and cotransfected with mimic NC. After 48 h of incubation, the cells were lysed using passive lysis buffer (Promega, Southampton, UK) and maintained at −80°C.

The protocol used in the cotransfection inhibition assays is the same protocol as the experiments mentioned above, replacing the corresponding miRNA mimics with inhibitors of that miRNA. In addition, an inhibitory negative control (NC-inh) was also employed in these experiments.

### 2.5. Statistical Analysis

The statistical analysis was performed using GraphPad Prism v.9.0 (GraphPad Software, CA, USA). Categorical variables are presented as percentages, and their odds ratios were calculated using the Chi-squared test. Welch's correction unpaired *t*-test was employed to compare the means of relative luciferase activity. A *p* value < 0.05 (2-sided) was considered statistically significant.

## 3. Results

### 3.1. Clinical and Genetic Characterization of the Patients

A total of 409 patients were included in the study. The main clinical features, LDL-c levels, and genetic testing results are summarized in Table 1. The patients with a confirmed genetic diagnosis were younger and had higher LDL-c levels than the patients without genetic confirmation. A family history of dyslipidemia was also statistically associated with a positive test. However, there was no positive association between carriers of pathogenic variants and a personal history of early CVD or a family history of CVD.

A total of 164 patients carried one or more variants classified as pathogenic, likely pathogenic, or variants of uncertain significance. Eighty-four percent of the variants were found in *LDLR*, 12% were found in *APOB*, and 4% were found in *PCSK9*.

### 3.2. Identification of *LDLR* and *PCSK9*-3′UTR Variants

**Figure 1:**
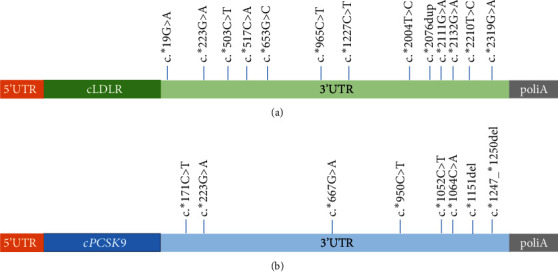
Schematic representation of the 3′UTR variants found in the patients: (a) *LDLR*-3′UTR variants; (b) *PCSK9*-3′UTR variants.

The extended analysis to the 3′-untranslated regions showed variants with frequencies less than 0.01 in 30 (7.3%) of the 409 patients. The main clinical and biochemical characteristics of these patients are shown in Table 2. The analysis of the 3′UTR region showed 13 variants at the *LDLR*-3′UTR in 14 patients and 8 variants at *PSCK9* in 16 patients (Figure 1). Eleven of the patients with 3′UTR variants already had a confirmed genetic diagnosis of FH. These patients were younger and had higher LDL-c levels than those without a confirmation.

We examined the phenotype of the 16 patients carrying only 3′UTR variants, and we observed that they showed a higher percentage of personal cardiovascular disease events compared with patients with confirmed genetic diagnosis (OR 4.5, *p* < 0.0065) (data in supplementary table SPTB2).

### 3.3. *In Silico* Prediction of the Effect of *LDLR* and *PCSK9*-3′UTR Variants on miRNA Binding

All *LDLR* and *PCSK9*-3′UTR variants with an allele frequency < 0.01 were analyzed with miRanda to predict miRNA binding. We realize an exhaustive analysis including all miRNA binding sites removed, modified, and added, as well as the analysis of the alignment with the sequences in order to select the most relevant 3′UTR variants for further studies. The criteria for selecting 3′UTR variants were miRanda prediction, variant allele frequency, and number of carriers. All relevant variants at *LDLR* and *PCSK9* are recorded in supplementary files (SPTB3). The *LDLR*-3′UTR variants which were predicted to create new illegitimate miRNA binding sites could decrease *LDLR* expression. The variants c.^∗^19G > A, c.^∗^503C > T, c.^∗^653G > C, c.^∗^1227C > T, c.^∗^2111G > A, and c.^∗^2319C > G were predicted to illegitimately bind to more than 10 miRNAs. Most of them could also remove or modify a miRNA binding site affecting the *LDLR* expression to some extent. Based on the larger number of predicted binding sites to miRNAs and the lower allele frequency, four *LDLR*-3′UTR variants were selected for functional validation by luciferase reporter assay: c.^∗^19G > A, c.^∗^503C > T, c.^∗^653G > C, and c.^∗^1227C > T. In addition, the c.^∗^517C > A variant was also included because it was carried by the same patient who also carried c.^∗^653G > C.

Five *PCSK9*-3′UTR variants were predicted to disrupt the binding sites of miRNAs: c.^∗^171C > T, c.^∗^234C > T, c.^∗^950C > T, c.^∗^1064C > A, and c.^∗^1151del. Two of these variants, c.^∗^171C > T and c.^∗^234C > T, stand out due to being carried by several patients. Furthermore, miRanda predicted that all variants could create new illegitimate miRNA binding sites.

The criteria employed to select *PCSK9*-3′UTR variants were a higher number of predicted miRNA binding sites removed, low allele frequency, and a higher frequency of the variants in the patients. Thus, c.^∗^171C > T, c.^∗^234C > T, and c.^∗^950C > T were selected for the functional study.

### 3.4. Functional Study to Evaluate the Impact of *LDLR*-3′UTR Variants

Luciferase activity of *LDLR *c.^∗^19G > A, c.^∗^503C > T, c.^∗^517C > A, c.^∗^653G > C, and c.^∗^1227C > T in HepG2.

**Figure 2:**
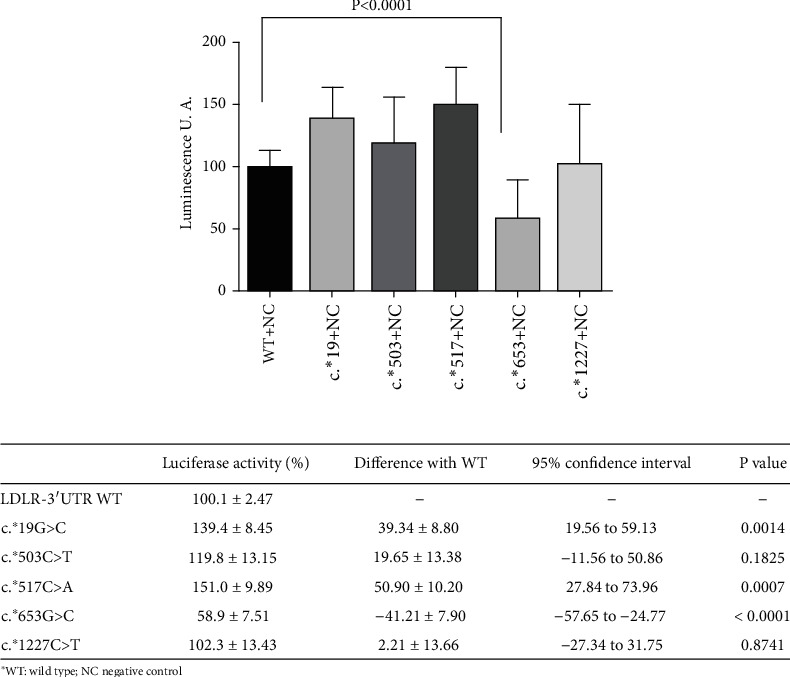
Comparison of the luciferase activity of LDLR-3′UTR variants using luciferase reporter assay. LDLR-3′UTR WT luciferase reporter construct or mutant-plasmids c.^∗^19G > A, c.^∗^503C > T, c.^∗^517C > A, c.^∗^653G > C, and c.^∗^1227C > T were transfected in HepG2 cells. The luciferase activity of cell lysates was then measured by chemiluminescence. Each result corresponds to the mean ± standard deviation of the triplicate assays of the luciferase activity of the LDLR-3′UTR WT luciferase reporter construct or mutant-plasmids: c.^∗^19G > A (c^∗^19), c.^∗^503C > T (c^∗^503), c.^∗^517C > A (c^∗^517), c.^∗^653G > C (c^∗^653), and c.^∗^1227C > T (c^∗^1227). Statistical significance was determined by a Welch's correction unpaired *t*-test (2-sided) and a 95% confidence interval. The results were obtained in three independent experiments.

The results of luciferase reporter assay of the *LDLR*-3′UTR constructs c.^∗^19G > A, c.^∗^503C > T, c.^∗^517C > A, c.^∗^653G > C, and c.^∗^1227C > T are shown in Figure 2. The variant c.^∗^653G > C led to a 41% decrease in luciferase expression when compared with the WT. This finding supports the creation of illegitimate new binding sites to miRNAs. The c.^∗^19G > A and c.^∗^517C > A constructs showed a significant increase in luciferase expression of up to 39% and 51%, respectively, compared with the WT, despite the fact that the *in silico* study predicted the creation of new illegitimate binding sites of several miRNAs. The c.^∗^503C > T and c.^∗^1227C > T constructs did not affect luciferase activity compared with the WT.

### 3.5. Selection of miRNAs for the Functional Characterization of *LDLR*-3′UTR Variants

Up to 65 miRNAs are predicted to illegitimately bind to the selected *LDLR*-3′UTR variants c.^∗^19G > A, c.^∗^503C > T, c.^∗^517C > A, c.^∗^653G > C, and c.^∗^1227C > T. Of these miRNAs, only miR-296-3p was predicted to bind to four variants—c.^∗^19G > A, c.^∗^503C > T, c.^∗^517C > A, and c.^∗^1227C > T—and was therefore selected for the experiments.

We analyzed 21 different miRNAs that could bind to c.^∗^653G > C. Nine of them can also bind *LDLR*-3′UTR WT according to the reanalysis with miRWalk, TargetScan, and miRBase (Table SPTB4). Of these miRNAs, miR-449c (which was related to control lipogenesis and cholesterogenesis in hepatoma cells [39]) was ultimately selected.

### 3.6. Cotransfection of miR-296-3p Mimic with *LDLR*-c.^∗^19G > A, c.^∗^503C > T, c.^∗^517C > A, and c.^∗^1227C > T

**Figure 3:**
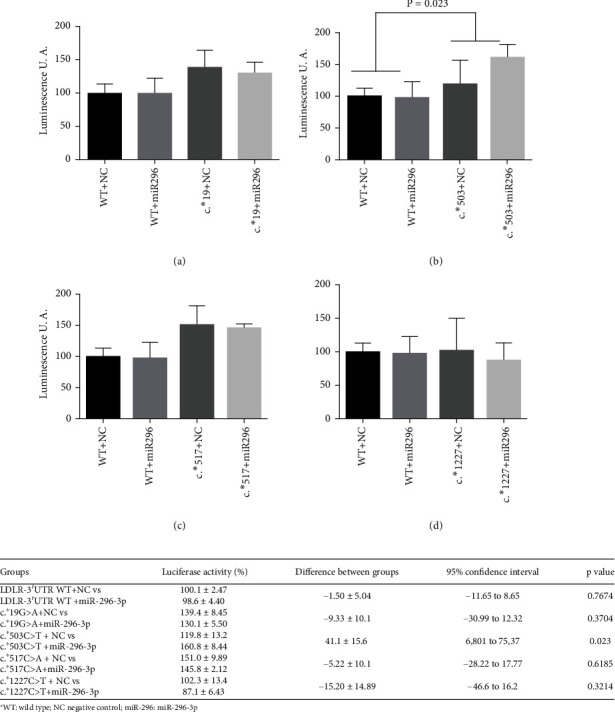
Effect of miR-296-3p on the expression of *LDLR*-3′UTR variants (a) c.^∗^19G > A, (b) c.^∗^503C > T, (c) c.^∗^517C > A, and (d) c.^∗^1227C > T in the luciferase reporter assay. 3′UTR-*LDLR* WT (WT) luciferase reporter construct or mutant-plasmids c.^∗^19G > A, c.^∗^503C > T, c.^∗^517C > A, c.^∗^653G > C, and c.^∗^1227C > T were cotransfected with miR-296-3p mimic or mimic negative control; the luciferase activity of the cell lysates was then measured. The luciferase activity of the variants cotransfected with miR-296-3p mimic or mimic negative control (CN) is shown in (a) c.^∗^19G > A (c^∗^19), (b) c.^∗^503C > T (c^∗^503), (c) c.^∗^517C > A (c^∗^517), and (d) c.^∗^1227C > T (c^∗^1227). Each result corresponds to the percentage of luciferase activity with respect to *LDLR*-3′UTR WT with mimic negative control ± standard deviation of the triplicate assays. Statistical significance was determined by a Welch's correction unpaired *t*-test (2-sided) and a 95% confidence interval. The results were obtained in three independent experiments.

The luciferase expression of *LDLR*:c.^∗^19G > A, c.^∗^503C > T, c.^∗^517C > A, and c.^∗^1227C > T cotransfected with miR-296-3p mimic is shown in Figure 3. Luciferase expression of c.^∗^19G > A, c.^∗^517C > A, and c.^∗^1227C > T cotransfected with miR-296-3p was weekly lower than mimic NC, but the difference was not statistically significant. We observed an increase in luciferase expression of c.^∗^503C > T cotransfected with miR-2963p compared with NC. This variant could increase *LDLR* expression instead of the expected effect of *LDLR* inhibition.

### 3.7. Cotransfection of miR-449c with *LDLR*:c.^∗^653G > T

**Figure 4:**
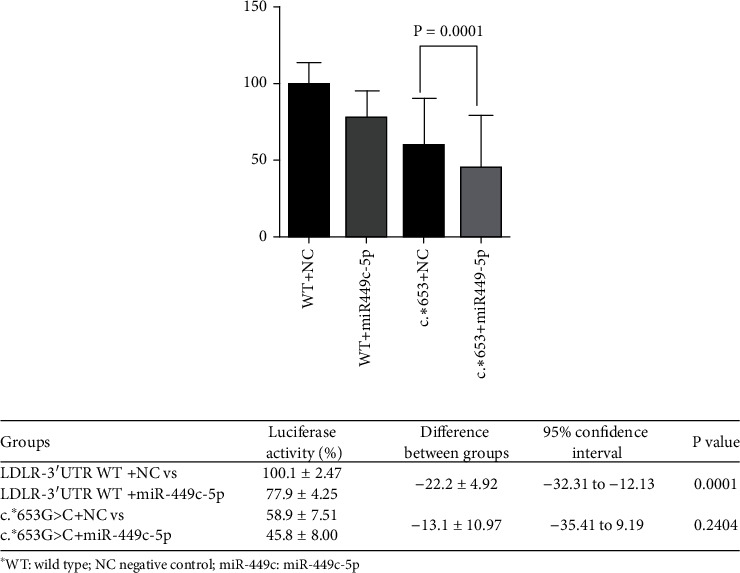
Effect of miR-449c-5p on the expression of *LDLR*-3′UTR variant c.^∗^653G > C in the luciferase reporter assay. The *LDLR*-3′UTR luciferase reporter construct WT (WT) and mutated-plasmid *LDLR*:c.^∗^653G > C (c^∗^653) were cotransfected with miR-449c-5p mimic or negative mimic control (CN). The luciferase activity of the cell lysates was then measured. The luciferase activity of the WT and mutant c.^∗^653G > C cotransfected with miR449c-5p or mimic negative control is shown. Each result corresponds to the percentage of luciferase activity with respect to WT with mimic negative control ± standard deviation of the triplicate assays. Statistical significance was determined by a Welch's correction unpaired *t*-test (2-sided) and a 95% confidence interval. The results were obtained in three independent experiments.

Results of the luciferase expression of *LDLR*:c.^∗^653G > C are shown in Figure 4. A decrease of up to 22% in luciferase expression between the cotransfection of 3′UTR-*LDLR* WT with miR-449c with respect to NC was observed, suggesting binding of miR-449c to 3′UTR-*LDLR* WT. The luciferase expression of c.^∗^653G > C cotransfected with miR-449c was lower than with the cotransfection with mimic NC, but the difference was not statistically significant.

### 3.8. Functional Study to Evaluate the Impact of the *PCSK9*-3′UTR Variants

Luciferase activity of *PCSK9*:c.^∗^171C > T, c.^∗^234C > T, and c.^∗^950C > T in HepG2.

**Figure 5:**
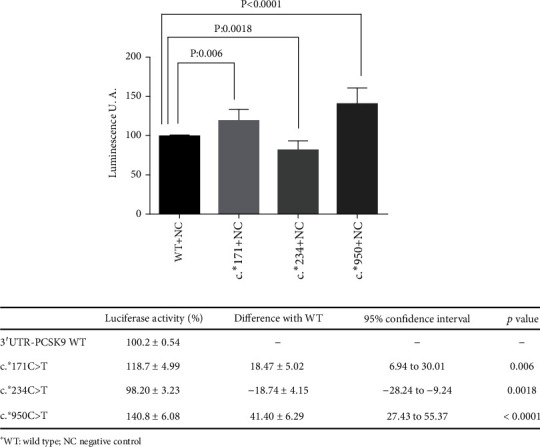
Comparison of the luciferase activity of 3′UTR-PCSK9 variants using the luciferase reporter assay. The 3′UTR-PCSK9 luciferase reporter construct WT (WT) or mutants c.^∗^171C > T (c.171), c.^∗^234C > T (c.234), and c.^∗^950C > T (c.950) were transfected in HepG2 cells. The luciferase activity of the cell lysates was then measured by luminescence. Each result corresponds to the mean ± standard deviation of the triplicate assays of the luciferase activity of the cell lysates of 3′UTR-PCSK9 luciferase reporter construct WT (WT) or mutants: c.^∗^171C > T (c.171), c.^∗^234C > T (c.234), and c.^∗^950C > T (c.950). Statistical significance was determined by a Welch's correction unpaired *t*-test (2-sided) and a 95% confidence interval. The results were obtained from three independent experiments.

The effect of the *PCSK9*-3′UTR variants on luciferase activity is shown in Figure 5. The luciferase expression of the c.^∗^171C > T and c.^∗^950C > T variants was 18% and 41% higher than that of *PCSK9* WT (*p* < 0.006 and *p* < 0.0018), which could indicate the removal of a miRNA binding site that downregulates *PCSK9* WT. These findings suggest the potential pathogenicity of these variants. Four patients carried c.^∗^171C > T, three of whom had no genetic confirmation.

In contrast, the luciferase expression of the c.^∗^234C > T experiment was 19% lower than that of *PCSK9* WT (*p* < 0.0001), an unexpected finding that could be caused by the creation of a new miRNA binding site despite the *in silico* predicted removal of an miRNA binding.

### 3.9. Selection of miRNAs for the Functional Characterization of *PCSK9*:c^∗^171C > T and c.^∗^234C > T

Based on miRanda, 10 miRNAs were predicted to be removed (Supplementary Table SPTB5). The selection of miRNAs was performed independently for each variant. The miRNAs predicted to be removed by c.^∗^171C > T and c.^∗^234C > T were validated with miRWalk3.0, TargetScan, and miRBase. miR-4269 was selected to test the variant c.^∗^171C > T, given that miRWalk3.0 and TargetScan predicted its binding to *PCSK9*-3′UTR. To test the variant c.^∗^234C > T, miR-1226-5p was chosen among the 4 miRNA candidates for the reporter luciferase assays.

### 3.10. Experiments on Overexpression of miR-4269 Mimic Cotransfected with *PCSK9*:c.^∗^171C > T

According to the miRanda prediction, c.^∗^171C > T removes an miR-4269 binding site. The luciferase expression of *PCSK9*:c.^∗^171C > T and *PCSK9* WT cotransfected with miR-4269 mimic did not show difference in the luciferase expression of *PCSK9* WT cotransfected with miR-4269 mimic or mimic NC, suggesting that *PCSK9* WT is not an miR-4269 target. However, the cotransfection of *PCSK9*:c.^∗^171C > T with miR-4269 showed a decrease of up to 20% compared with mimic NC, which was statistically significant (*p* < 0.004), a finding that suggests that c.^∗^171C > T might enhance miR-4269 binding (SF1a).

### 3.11. Experiments on Overexpression of miR-1226-5p Mimic Cotransfected with *PCSK9*:c.^∗^234C > T

These experiments tested the binding of miR-1226-5p with *PCSK9* WT and removal of that binding site with *PCSK9*:c.^∗^234C > T. An unexpected and statistically significant increase of up to 41% was observed in the luciferase expression of *PCSK9* WT cotransfected with miR-1226-5p compared with mimic NC, a result contrary to the expected repression due to the binding between miRNA and *PCSK9* WT.

There was no difference between the luciferase expression of *PCSK9*:c.^∗^234C > T cotransfected with miR-1226-5p or the mimic NC. In the presence of the variant, the effect of miRNA on increasing luciferase expression was not observed (SF1b).

### 3.12. Experiments on Inhibition with Anti-miR-1226-5p Inhibitor Cotransfected with *PCSK9*:c.^∗^234C > T

The miR-1226-5p overexpression experiments suggested that the miRNA could upregulate *PCSK9*. Subsequently, an inhibition experiment with an anti-miR-1226-5p inhibitor was performed to reverse the miRNA effect.

The luciferase expression in the experiments of cotransfection of *PCSK9* WT with inhibitor or inhibitor NC negative control (NC-inh) is shown in SF2. Cotransfection of *PCSK9* WT with inhibitor decreased luciferase expression by up to 12% compared with NC-inh but was not statistically significant, a result that could be due to the inhibition of miRNA-1226-5p. In contrast, the luciferase expression of the cotransfection of *PCSK9*:c.^∗^234C > T with inhibitor was 20% higher than that of the NC-inh but was not statistically significant.

## 4. Discussion

In this study, we evaluated the impact of 3′UTR variants on the FH diagnosis of 406 patients with hypercholesterolemia. We first performed a routine molecular FH test that included studying the exonic, adjacent intronic, and promoter regions of the main genes *LDLR*, *APOB*, *PCSK9*, and *LDLRAP1* and then extended the analysis to the 3′UTR-*LDLR* and *PCSK9* regions. Routine genetic FH testing showed a causative variant (positive test) in 164 patients, while 245 patients had a negative test. The test was similar to that reported by other groups [11–14]. The performance of the genetic test can be related to the clinical criteria employed in the FH diagnosis. LDL-c levels, a family history of dyslipidemia, and age (younger) showed a strong association with a positive test, while there was no association with a personal or family history of CVD. Extremely high LDL-c levels have been associated with the presence of pathogenic variants and are the main feature of familial hypercholesterolemia [4, 14, 40]. A family history of dyslipidemia and elevated LDL-c levels in younger patients is also associated with FH. However, an association between patients with CVD and pathogenic variants was not observed in our study, despite the fact that the risk of CAD is higher than in carriers [4]. Our result could be due to the fact that for most of our patients, the genetic test was requested based on a personal and/or family history of CVD according to cardiovascular risk guidelines [41], but only 2% of the patients with LDL-c levels > 190 mg/dL carried causative genetic variants [4]. Indeed, many of our patients with a personal or family history of CVD had LDL-c levels < 250 mg/dL, and a high percentage of them had a negative test. Furthermore, finding causative variants in this patient group would improve the diagnosis and clinical management and allow cascade screening in family members.

The analysis of the complete *LDLR* and *PCSK9*-3′UTR regions in our patients showed a low frequency of 3′UTR variants in 30 of the patients (7%): 13 variants in *LDLR*-3′UTR and 8 variants in *PCSK9*-3′UTR. Variants in *LDLR*-3′UTR could cause hypercholesterolemia by removing miRNA binding, while *PCSK9*-3′UTR variants could upregulate *PCSK9* by adding illegitimate binding sites. Several studies have indicated the relevance of LDL-c levels in miRNA-mediated regulation in the expression of *PCSK9* and *LDLR* by targeting their 3′UTR regions [24, 25, 28, 42], which could be severely altered by 3′UTR variants.

To predict the 3′UTR variant effect, we performed an *in silico* analysis, the results of which showed that all *LDLR*-3′UTR variants could have a pathogenic effect by creating illegitimate miRNA binding. In addition, 5 of the 8 *PCSK9*-3′UTR variants were predicted to remove miRNA binding sites and have potentially pathogenic variants. Previous studies have shown that *LDLR*:c.^∗^504 A>G and c.^∗^773 A>G variants associated with low HDL levels and a higher risk of CVD could downregulate LDLR by illegitimate binding with miR-200a and miR-638, according to *in silico* predictions [30]. In another study, a bioinformatics analysis of seven 3′UTR variants in *PCSK9* carried by Brazilian patients with FH predicted the removal of miRNA binding that could upregulate *PCSK9* [19]. The functional characterization of this class of variants is necessary because the dual behavior (adding and removing miRNA binding) is often predicted, and its impact should be confirmed.

Among the 3′UTR-LDLR variants tested in this study, *LDLR*:c.^∗^653G > C demonstrated the largest impact, decreasing luciferase expression by 41% compared with LDLR WT. According to the miRanda prediction, this variant could add a high number of illegitimate miRNA binding sites. As far as we know, however, none of these miRNAs have been previously reported in association with FH. miRNA-449c was selected among the 21 miRNA-added candidates to test its differential binding with *LDLR*:c.^∗^653G > C and LDLR WT. This miRNA had been associated with lipogenesis and cholesterogenesis control in hepatocarcinoma cells through the inhibition of SIRT1 and SREBP-1c expression and downregulation of their targeted genes, including fatty acid synthase and 3-hydroxy-3-methylglutaryl CoA reductase [39]. We hypothesized that illegitimate binding with *LDLR*:c.^∗^653G > C could dysregulate the cholesterol pathway. *In silico* analysis predicted miRNA-449c binding to LDLR WT, which agreed with the result of the luciferase reporter assay that showed a 20% decrease in luciferase expression. However, miRNA449c binding with c.^∗^653G > C was not confirmed. The 41% decrease in LDLR expression observed in the above experiment could be due to one or more miRNAs other than miR449c. The genetic diagnosis in patient 2, a carrier of c.^∗^653G > C, was not confirmed in a routine test. This patient had a characteristic clinical phenotype of familial hypercholesterolemia, with high LDL-c levels (195–250 mg/dL), a personal history of early acute myocardial infarction, and a family history of cardiovascular disease, which also suggest a possible pathogenic effect of this variant.

The variants, *LDLR*:c.^∗^19G > A and c.^∗^517C > A, showed an increase in luciferase expression compared with LDLR WT. These variants could add and remove miRNA binding sites based on the miRanda analysis, but only the latter effect was observed in the experiments. These variants could therefore have a gain-of-function effect. In our study, patient 9 with c.^∗^19G > A also carried the heterozygous pathogenic variant *LDLR*:c.2416dupG: p.(Val806Glufs^∗^11). This patient had very high LDL-c levels (250–329 mg/dL), skin manifestations of hypercholesterolemia (such as xanthomas), and a family history of hypercholesterolemia, characteristics of a heterozygous but not homozygous phenotype. The effect of the c.^∗^19 variant was gain-of-function according to the functional analysis; however, its impact was insufficient to counteract the effect of the pathogenic variant c.2416dupG: p.(Val806Glufs^∗^11).

Patient 2 with c.^∗^517C > A also had the c.^∗^653G > C variant and had high LDL-c levels (190–249 mg/dL). The gain-of-function effect of c.^∗^517C > A observed in the functional study does not explain the phenotype; the presence of c.^∗^653G > C is more likely the cause of the increases in LDL-c. Previous studies have shown that 3′UTR-LDLR variants that were more frequently observed in patients with hypercholesterolemia than controls also showed an increase in luciferase expression, with these variants ultimately being classified as benign or protective [20].

*LDLR*:c.^∗^503C > T and c.^∗^1227C > T showed no differences in luciferase activity compared with WT. These variants would not create effective new miRNA binding sites and would be benign. It is possible that the affected miRNAs would have a very low concentration in HepG2 and a nonmeasurable repression effect. Patient 1 with c.^∗^503C > T had no genetic diagnosis but had high LDL-c levels (190–249 mg/dL) and a family history of dyslipidemia and CVD. Polygenic hypercholesterolemia or other undescribed variants could explain the patient's phenotype. Patient 11 with c.^∗^1227C > T also carried the likely pathogenic variant *LDLR*:c.1118G>A: p.(Gly373Asp) in heterozygosity, which could explain the clinical features.

The impact of 3′UTR variants also depends on the presence of miRNAs and their levels in the cells. The variants c.^∗^19G > A, c.^∗^503C > T, c.^∗^517C > A, and c.^∗^1227C > T were cotransfected with miR-296-3p, given that the *in silico* analysis predicted an illegitimate binding with these variants. miR-296 has been related to the pathogenesis of atherosclerosis and has been associated with angiogenesis, inflammatory response, and cholesterol metabolism [43].

The miR-296-3p overexpression experiments showed that LDLR 3′UTR WT is not a target of this miRNA or of c.^∗^19G > A, c.^∗^503C > T, c.^∗^517C > A, and c.^∗^1227C > T. The variant c.^∗^503C > T showed an unexpected increased luciferase expression when it was cotransfected with miR-296-3p, suggesting an effect of removing miRNA binding. However, the other three variants, c.^∗^19G > A, c.^∗^517C > A, and c.^∗^1227C > T, showed a decrease in luciferase expression when they were cotransfected with the miRNA, but the decrease was not statistically significant. These differences could be due to weak miRNA-variant binding and have a similar value as that reported in previous studies with other 3′UTR variants [29]. In addition, the bioinformatics tool used for the prediction (miRanda) has notable sensitivity [44] and predicts weak miRNA binding that would be difficult to test experimentally.

The PCSK9 protein acts as a posttranscriptional LDLR expression regulator; the gain-of-function variants at *PCSK9* are associated with hypercholesterolemia due to the effect on LDLR degradation. Among the *PCSK9*-3′UTR variants found in the patients, 5 of them could remove miRNA binding sites, suggesting a gain-of-function effect. Three underwent a functional study: c.^∗^171C > T, c.^∗^234C > T, and c.^∗^950C > T. *PCSK9*:c.^∗^171C > T showed an increase in luciferase expression and could cause upregulated PCSK9 expression. Cotransfection with miR-4269 was performed to test the *in silico* prediction of miRNA binding removal by this miRNA. However, the *in silico* prediction was not confirmed because the miR-4269 showed no binding with the WT. It is therefore unlikely that this miRNA is related to the gain-of-function effect observed in c.^∗^171C > T. Accordingly, with the observed decrease in luciferase expression, c.^∗^171C > T could create a new binding site with miR-4269. This unexpected result, which is in contrast to the *in silico* prediction, supports the importance of the functional validation of these variants, as already reported in the miRNA binding between hsa-miR-1228-3p and the variant *PCSK9*:c.^∗^571C > T [29]. The high number of carriers of this variant in our patient group also supports its possible pathogenic effect. This variant was recently observed to have an 8-fold higher allelic frequency in Brazilian patients with hypercholesterolemia than in the general population [19]. The analysis of the clinical phenotype of patients 15, 16, 17, and 28 who were carriers of c.^∗^171C > T suggests a moderate hypercholesterolemic effect. Three of the patients showed high but not very high LDL-c levels (190–250 mg/dL), and one of them also carried another pathogenic variant (APOB p.(Arg3527Gln)). This patient was young (21 years old), and their LDL-c levels might increase with aging. Patient 17 had a definitive clinical diagnosis of FH and the most severe phenotype with LDL-c>329 but no genetic confirmation. This interesting patient could carry a variant not detected in our genetic analysis. Also, two patients had a personal history of CVD but not particularly high LDL-c levels (190–250 mg/dL).

The functional characterization of *PCSK9*:c.^∗^950C > T showed that this variant could also cause hypercholesterolemia by PCSK9 upregulation according to the observed increase in luciferase expression (up to 41%). The extremely low allelic frequency (0.000095 according to Gnomad) also suggests the variant's possible pathogenic impact. The *in silico* study predicted that this variant could remove the binding site of several miRNAs in PCSK9 WT. The clinical phenotype of patient 21 (a carrier of c.^∗^950C > T, with no genetic confirmation, very high LDL-C levels (250–329 mg/dL), and a family history of hypercholesterolemia) is characteristic of familial hypercholesterolemia and supports the pathogenic effect of the variant. Further studies are recommended to clarify this variant's mechanism of action.

Lastly, the *PCSK9*:c.^∗^234C > T variant was observed at a high rate (1.4%) in our patient group. This variant had already been reported with a higher allelic frequency in patients with hypercholesterolemia than in the general population [19]. The variant's *in silico* analysis predicted the removal of several miRNA binding sites that could justify the observed hypercholesterolemia through a mechanism based on PCSK9 upregulation. However, the *in silico* analysis also predicted the creation of new illegitimate miRNA binding sites. Accordingly, with the decrease in luciferase expression observed in the experiment, this variant could have a loss-of-function effect. This result highlights the importance of in vitro studies for characterizing 3′UTR variants, as has already been observed in other common LDLR 3′UTR variants with an increased prevalence in patients with hypercholesterolemia but showing a functional protective effect [20].

The effect of *PCSK9*:c.^∗^234C > T on PCSK9 expression could depend on the miRNA concentration; to elucidate this, we performed an miRNA overexpression experiment. We chose miR-1226-5p because the *in silico* study predicted a target in *PCSK9*-3′UTR, which is removed by c.^∗^234C > T. However, the in vitro studies showed unexpected results. Cotransfection of *PCSK9*-3′UTR with miR-1226-5p showed *PCSK9*-3′UTR upregulation according to the observed increase in luciferase expression, which was removed when the miRNA was cotransfected with c.^∗^234C > T. In these experiments, miR-1226-5p had an apparent promotor effect instead of a repressive effect associated with miRNAs, which is not be observed with c.^∗^234C > T due to the removal of miRNA binding. A number of studies have reported a rare promotor effect in the miRNAs [45, 46]. An alternative mechanism would be that miR-1226-5p and the c.^∗^234C > T variant had two independent effects: perhaps, the miRNA miR-1226-5p downregulated an inhibitor of luciferase reporter, such as another miRNA or another gene, and thereby indirectly upregulated the luciferase reporter, while c.^∗^234C > T created a new miRNA binding site and downregulated the luciferase reporter.

We also wanted to retest the effect of miR-1226-5p on PCSK9 with an inhibitor overexpression experiment. The miR-1226-5p inhibitor showed a slight decrease in luciferase expression when cotransfected with PCSK9 WT, which is consistent with the previous experiment. Conversely, miR-1226-5p inhibitor cotransfected with c.^∗^234C > T showed increased luciferase expression. Inhibition experiments appear to support the miR-1226-5p promotor effect, but the results were not statistically significant. There are currently no studies that have related miR-1226-5p to PCSK9, and more studies are needed to clarify the role of miR-1226-5p as an in vivo promoter of PCSK9 expression.

## 5. Conclusions

The genetic diagnosis of patients with suspected FH could be improved by extending the analysis to the 3′UTR regions of the main genes associated with FH, such are *LDLR* and *PCSK9*. In our study, 30 of the 409ç patients carried low-frequency variants in these regions, several of which could have pathogenic potential according to the *in silico* and functional studies. The results of this study are promising, although they need to be validated because determining the pathogenicity of variants in 3′UTR is a complex process that includes their interaction with miRNAs.

## Figures and Tables

**Table 1:** Main clinical characteristics of patients and results of genetic testing.

		Positive genetic test	Negative genetic test	Odds ratio	*p* value
Total patients	409	164 (40.1%)	245 (59.9%)		

Sex	Female Male	91 73	144 101	0.874	*p* = 0.5099

Age (years)		40.4 ± 18.3	50.3 ± 14.3		*p* < 0.0001

LDL-c (mg/dL)	>250 <250	96 55	85 149	3.060	*p* < 0.0001

Family history of dyslipemia	Yes No	126 23	171 57	1.826	*p* = 0.0264

Family history of CVD/CAD	Yes No	79 70	148 81	0.618	*p* = 0.0243

Personal history of CVD/CAD	Yes No	22 128	54 176	0.560	*p* = 0.0358

Genetic variants			*n* (%)		
*LDLR*			138 (84%)		
*APOB*			19 (12%)		
*PCSK9*			7 (4%)		

CVD/CAD: cardiovascular disease/coronary artery disease. Note: Data of LDL-c was available in 385 (94%); of family history of dyslipidemia, in 378 (92%); of family history of CVD/CAD, in 377 (92%); and of personal history of CVD/CAD in 380 (93%).

**Table 2:** Clinical and biochemical characteristics of patients with *LDLR* and *PCSK9*-3′UTR variants.

ID	Sex	Age	DLCNS FH	Familial history			Physical examination	Personal history	LDL-c (mg/dL)	Canonic variants; ACMG classification	3′UTR variants
				CADCVD/high LDL-c	Xanthomas early corneal arcus	High LDL-c (<15 y)	Xanthomas early corneal arcus	Early CAD/CPVD			
1	F	69	PB	Yes/yes	No	No	No/no	No/no	190-249		*LDLR*:c.^∗^503C > T
2	M	56	PB	No/yes	No	No	No/no	Yes/no	190-249		***LDLR*: ** **c**.^∗^517**C** > **A**;***LDLR*:c. **^∗^653**G** > **C**
3	F	62	PB	Yes/no	No	No	No/no	Yes/no	190-249		*LDLR*:c.^∗^965C > T
4	M	59	PB	Yes/yes	No	Yes	No/no	Yes/no	155-189		*LDLR*:c.^∗^2111G > A
5	F	60	PS	Yes/yes	No	No	No/no	No/no	190-249		*LDLR*:c.^∗^2132G > A
6	F	32	UP	No/yes	No	No	No/no	No/no	155-189		*LDLR*:c.^∗^2210T > C
7	F	56	PB	Yes/yes	No	Yes	No/no	No/no	250-329		*LDLR*:c.^∗^2319C > G
8	F	64	PB	Yes/no	No	No	No/no	No/no	250-329		*LDLR*:c.^∗^2319C > G
9^$^	M	38	D	Yes/yes	No	No	Yes/yes	No/no	250-329	*LDLR*:c.2416dupG: p.(Val806Gglyfs^∗^11); P	***LDLR*: ** **c**.^∗^19**G** > **A**
10^$^	F	34	PB	No/yes	No	Yes	No/no	No/no	190-249	*LDLR*:c.2043C>A p.(Cys681Ter); P	*LDLR*:c.^∗^223G > A
11^$^	M	7	PB	No/yes	No	Yes	No/no	No/yes	190-249	*LDLR*: c.1118G>A: p.(Gly373Asp); LP	*LDLR*:c.^∗^1227C > T
12^$^	F	77	D	No/no	No	No	Yes/no	No/no	250-329	*LDLR*: c.97C > T: p.(Gln33Ter); P	*LDLR*:c.^∗^2004C > T
13^$^	M	57	PB	No/no	No	Yes	No/no	Yes/no	190-249	*LDLR*: c.1951G>T: p.(Asp651Tyr); P	*LDLR*:c.^∗^2076dup
14	M	55	PB	No/yes	No	No	No/no	Yes/no	190-249	*APOB:* c.7241A>T: p.(Glu2414Val); VUS	*LDLR*:c.^∗^2210T > C
15	F	47	PS	No/no	No	No	No/no	Yes/no	190-249		***PCSK9*: ** **c**.^∗^171**C** > **T**
16	F	54	D	Yes/yes	No	Yes	No/no	No/no	>329		***PCSK9*: ** **c**.^∗^171**C** > **T**
17	F	12	PS	Yes/no	No	No	No/no	No/no	190-249		***PCSK9*: ** **c**.^∗^171**C** > **T**
18	M	59	D	Yes/yes	No	No	No/no	Yes/yes	250-329		***PCSK9*: ** **c**.^∗^234**C** > **T**
19	M	9	PB	No/yes	No	No	No/no	Yes/no	190-249		***PCSK9*: ** **c**.^∗^234**C** > **T**
20	F	63	PB	No/yes	No	No	No/no	No/yes	250-329		***PCSK9*: ** **c**.^∗^234**C** > **T**; *PCSK9*:c.^∗^1052C > T
21	F	64	PB	No/yes	No	No	No/no	No/no	250-329		***PCSK9*: ** **c**.^∗^950**C** > **T**
22	M	40	PB	Yes/yes	No	No	No/no	No/no	190-249		*PCSK9*:c.^∗^1052C > T
23^$^	F	50	PB	No/yes	No	No	No/no	No/no	250-329	*LDLR*:c.851G>A: p.(Cys284Tyr); LP	***PCSK9*: ** **c**.^∗^234**C** > **T**
24^$^	F	46	D	Yes/yes	No	Yes	No/yes	No/no	190-249	*LDLR*:c.1775G>A: p.(Gly592Glu); P	***PCSK9*: ** **c**.^∗^234**C** > **T**
25^$^	M	18	D	Yes/yes	No	No	No/no	No/no	>329	*LDLR*:c.2093G>A: p.Cys698Tyr; LP *PCSK9*:c.1633G>A: p.(Ser545Gly); VUS	*PCSK9*:c.^∗^667G > A
26^$^	F	75	PB	No/no	No	No	No/no	No/no	>329	*LDLR*:c.590G>A: p.(Cys197Tyr); P	*PCSK9*:c.^∗^1064C > A
27^$^	F	57	D	No/yes	No	Yes	No/yes	No/no	>329	*LDLR*:c.1358 + 1G > A; P	*PCSK9*:c.^∗^1151del
28^$^	M	21	PS	No/yes	No	No	No/no	No/no	190-249	*APOB*:c.10580G>A: p.(Arg3527Gln); P	***PCSK9*: ** **c**.^∗^171**C** > **T**
29	F	41	PS	Yes/no	No	No	No/no	No/no	190-249	*APOB*:c.11401T>A: p.(Ser3801Thr); VUS	***PCSK9*: ** **c**.^∗^234**C** > **T**
30	M	55	PB	No/yes	No	No	No/no	Yes/no	190-249	*APOB*:c.7241A>T: p.(Glu2414Val); VUS	*PCSK9*: c.^∗^1247_∗1250del

^$^Patients with genetic diagnostic confirmed. Abbreviations: ID: patient identification; FH: familial hypercholesterolemia; DLCNS: Dutch Lipid Clinic Network. According with the DCLN score, the clinical diagnosis of FH can be categorized as follows: D: definite; PB: probable; PS: possible; UP: unlikely; CVD: cardiovascular disease; CAD: coronary artery disease; CPVD: cerebral or peripheral vascular disease; CADD: combined annotation-dependent depletion (benign < 11); ACMG: American College of Medical Genetics classification; P: pathogenic; LP: likely pathogenic; VUS: variant of uncertain significant. Note: Variants in bold means that they are carried by more than one patient.

## Data Availability

Data is available on request.

## Supplementary Materials

Supplementary 1 Table SPTB1: summary of the regions captured in the custom panel of genes used in the patients' genetic testing.

Supplementary 2 Table SPTB2: comparison of the phenotype of patients with only 3′UTR variants with patients with and without confirmed genetic diagnosis and comparison of patients with 3′UTR variants with patients with confirmed genetic diagnosis.

Supplementary 3 Table SPTB3: 3′*UTR-LDLR* and 3′*UTR-PCSK9* variants selected after considering the effect on miRNA binding according to miRanda prediction, allele frequency, and number of carriers. Note: Variants in bold were selected for further functional characterization.

Supplementary 4 Table SPTB4: validation of the predictions made by the miRanda algorithm on the added miRNA binding sites due to the 3′UTR-*LDLR* variant c.^∗^653G > C with three other bioinformatics tools (miRWalk3.0, TargetScan, and miRDB).

Supplementary 5 Table SPTB5: validation of the predictions made by the miRanda algorithm on removed miRNA binding sites due to the following 3′UTR-*PCSK9* variants: c.^∗^171C > T, c.^∗^234C > T, and c.^∗^950C > T, with three other bioinformatics tools (miRWalk3.0, TargetScan, and miRDB).

Supplementary 6 Figure SF1: (a) effect of miR-4269 on the expression of the 3′UTR-*PCSK9* variant c.^∗^171C > T and (b) effect of miR-1226 on the expression of the 3′UTR-*PCSK9* variant: c.^∗^234C > T in the luciferase reporter assay.

Supplementary 7 Figure SF2: effect of miR-1226 inhibitor on the expression of the 3′UTR-PCSK9 variant c.^∗^234C > T versus 3′UTR-*PCSK9* WT in the luciferase reporter assay.
