# Supplementary material for: Functional Analysis of 3′UTR Variants at the LDLR and PCSK9 Genes in Patients with Familial Hypercholesterolemia
Source: Hum Mutat. 2024 Feb 8;2024:9964734. doi: 10.1155/2024/9964734 (PMC11918801; doi:10.1155/2024/9964734)
Supplement: Supplementary 1 — Table SPTB1: summary of the regions captured in the custom panel of genes used in the patients' genetic testing. [file 9964734.f1.docx]

**Table SPTB1**. Summary of the regions captured in the custom panel of genes used in the patients’ genetic testing.

| **Gene** | **Refseq** | **Exon** | **Chr** | **Start** | **End** | **Exon length** |
| --- | --- | --- | --- | --- | --- | --- |
| LDLR | NM_000527 | exon1 | chr19 | 11200224 | 11200291 | 67 |
| LDLR |  |  | chr19 | 11201264 | 11201605 | 341 |
| LDLR | NM_000527 | exon2 | chr19 | 11210898 | 11211021 | 123 |
| LDLR | NM_000527 | exon3 | chr19 | 11213339 | 11213462 | 123 |
| LDLR | NM_000527 | exon4 | chr19 | 11215895 | 11216276 | 381 |
| LDLR | NM_000527 | exon5 | chr19 | 11217240 | 11217363 | 123 |
| LDLR | NM_000527 | exon6 | chr19 | 11218067 | 11218190 | 123 |
| LDLR | NM_000527 | exon7 | chr19 | 11221327 | 11221447 | 120 |
| LDLR | NM_000527 | exon8 | chr19 | 11222189 | 11222315 | 126 |
| LDLR | NM_000527 | exon9 | chr19 | 11223953 | 11224125 | 172 |
| LDLR | NM_000527 | exon10 | chr19 | 11224210 | 11224438 | 228 |
| LDLR | NM_000527 | exon11 | chr19 | 11226769 | 11226888 | 119 |
| LDLR | NM_000527 | exon12 | chr19 | 11227534 | 11227674 | 140 |
| LDLR | NM_000527 | exon13 | chr19 | 11230767 | 11230909 | 142 |
| LDLR | NM_000527 | exon14 | chr19 | 11231045 | 11231198 | 153 |
| LDLR | NM_000527 | exon15 | chr19 | 11233849 | 11234020 | 171 |
| LDLR | NM_000527 | exon16 | chr19 | 11238683 | 11238761 | 78 |
| LDLR | NM_000527 | exon17 | chr19 | 11240188 | 11240346 | 158 |
| LDLR | NM_000527 | exon18 | chr19 | 11241956 | 11241992 | 36 |
| PCSK9 | NM_174936 | exon1 | chr1 | 55505510 | 55505717 | 207 |
| PCSK9 | NM_174936 | exon2 | chr1 | 55509515 | 55509707 | 192 |
| PCSK9 | NM_174936 | exon3 | chr1 | 55512195 | 55512319 | 124 |
| PCSK9 |  |  | chr1 | 55516854 | 55516969 | 115 |
| PCSK9 | NM_174936 | exon4 | chr1 | 55517950 | 55518084 | 134 |
| PCSK9 | NM_174936 | exon5 | chr1 | 55518322 | 55518464 | 142 |
| PCSK9 | NM_174936 | exon6 | chr1 | 55521665 | 55521862 | 197 |
| PCSK9 | NM_174936 | exon7 | chr1 | 55523003 | 55523187 | 184 |
| PCSK9 | NM_174936 | exon8 | chr1 | 55523708 | 55523882 | 174 |
| PCSK9 | NM_174936 | exon9 | chr1 | 55524171 | 55524320 | 149 |
| PCSK9 | NM_174936 | exon10 | chr1 | 55525158 | 55525336 | 178 |
| PCSK9 | NM_174936 | exon11 | chr1 | 55527047 | 55527229 | 182 |
| PCSK9 | NM_174936 | exon12 | chr1 | 55529041 | 55529257 | 216 |
| APOB | NM_000384 | exon29 | chr2 | 21224601 | 21226206 | 1605 |
| APOB | NM_000384 | exon28 | chr2 | 21227140 | 21227324 | 184 |
| APOB | NM_000384 | exon27 | chr2 | 21227432 | 21227547 | 115 |
| APOB | NM_000384 | exon26 | chr2 | 21227951 | 21235523 | 7572 |
| APOB | NM_000384 | exon25 | chr2 | 21236031 | 21236405 | 374 |
| APOB | NM_000384 | exon24 | chr2 | 21237319 | 21237465 | 146 |
| APOB | NM_000384 | exon23 | chr2 | 21237944 | 21238132 | 188 |
| APOB | NM_000384 | exon22 | chr2 | 21238241 | 21238417 | 176 |
| APOB | NM_000384 | exon21 | chr2 | 21239310 | 21239521 | 211 |
| APOB | NM_000384 | exon20 | chr2 | 21241863 | 21241985 | 122 |
| APOB | NM_000384 | exon19 | chr2 | 21242594 | 21242777 | 183 |
| APOB | NM_000384 | exon18 | chr2 | 21245702 | 21245914 | 212 |
| APOB | NM_000384 | exon17 | chr2 | 21246396 | 21246564 | 168 |
| APOB |  |  | chr2 | 21247655 | 21247758 | 103 |
| APOB | NM_000384 | exon16 | chr2 | 21247804 | 21247996 | 192 |
| APOB | NM_000384 | exon15 | chr2 | 21249659 | 21249836 | 177 |
| APOB | NM_000384 | exon14 | chr2 | 21250699 | 21250937 | 238 |
| APOB | NM_000384 | exon13 | chr2 | 21251198 | 21251410 | 212 |
| APOB | NM_000384 | exon12 | chr2 | 21252510 | 21252657 | 147 |
| APOB | NM_000384 | exon11 | chr2 | 21252769 | 21252887 | 118 |
| APOB | NM_000384 | exon10 | chr2 | 21255225 | 21255453 | 228 |
| APOB | NM_000384 | exon9 | chr2 | 21256170 | 21256390 | 220 |
| APOB | NM_000384 | exon8 | chr2 | 21257687 | 21257773 | 86 |
| APOB | NM_000384 | exon7 | chr2 | 21258455 | 21258580 | 125 |
| APOB | NM_000384 | exon6 | chr2 | 21259971 | 21260127 | 156 |
| APOB | NM_000384 | exon5 | chr2 | 21260829 | 21260983 | 154 |
| APOB | NM_000384 | exon4 | chr2 | 21263809 | 21263955 | 146 |
| APOB | NM_000384 | exon3 | chr2 | 21265232 | 21265348 | 116 |
| APOB | NM_000384 | exon2 | chr2 | 21266384 | 21266423 | 39 |
| APOB | NM_000384 | exon1 | chr2 | 21266735 | 21266817 | 82 |
| LDLRAP1 | NM_015627 | exon1 | chr1 | 25870189 | 25870277 | 88 |
| LDLRAP1 | NM_015627 | exon2 | chr1 | 25880412 | 25880555 | 143 |
| LDLRAP1 | NM_015627 | exon3 | chr1 | 25881350 | 25881463 | 113 |
| LDLRAP1 | NM_015627 | exon4 | chr1 | 25883643 | 25883758 | 115 |
| LDLRAP1 | NM_015627 | exon5 | chr1 | 25889134 | 25889207 | 73 |
| LDLRAP1 | NM_015627 | exon6 | chr1 | 25889560 | 25889644 | 84 |
| LDLRAP1 | NM_015627 | exon7 | chr1 | 25890151 | 25890282 | 131 |
| LDLRAP1 | NM_015627 | exon8 | chr1 | 25891663 | 25891698 | 35 |
| LDLRAP1 | NM_015627 | exon9 | chr1 | 25893338 | 25893483 | 145 |
