# Supplementary material for: Functional Analysis of 3′UTR Variants at the LDLR and PCSK9 Genes in Patients with Familial Hypercholesterolemia
Source: Hum Mutat. 2024 Feb 8;2024:9964734. doi: 10.1155/2024/9964734 (PMC11918801; doi:10.1155/2024/9964734)
Supplement: Supplementary 2 — Table SPTB2: comparison of the phenotype of patients with only 3′UTR variants with patients with and without confirmed genetic diagnosis and comparison of patients with 3′UTR variants with patients with confirmed genetic diagnosis. [file 9964734.f2.docx]

**Table SPTB2.** Comparison of the phenotype of patients with only 3'UTR variants with patients with and without confirmed genetic diagnosis; and, comparison of patients with 3'UTR variants with patients with confirmed genetic diagnosis.

|  |  | **Negative genetic test** | **3'UTR only** | Odds ratio (negative test) | *p value* | **Positive genetic test** | **3'UTR only** | Odds ratio (positive test) | *p value* | **Positive genetic test** | **3'UTR + other variant** | Odds ratio (positive test) | *p value* |
| --- | --- | --- | --- | --- | --- | --- | --- | --- | --- | --- | --- | --- | --- |
| **Total patients** |  | 245 (59.9%) | 16 |  |  | 164 (40,1%) | 16 |  |  | 164 (40,1%) | 14 |  |  |
| **Sex** | **female** | 144 | 11 | 1,5431 | *0.4343* | 91 | 11 | 1,7648 | *0.3119* | 91 | 8 | 1,0696 | *0.9048* |
|  | **male** | 101 | 5 |  |  | 73 | 5 |  |  | 73 | 6 |  |  |
| **Age (years)** |  | 50,3 ± 14,3 | 50,4 ± 18,21 | - | *0,9934* | 40,4 ± 18,3 | 50,4 ± 18,21 | - | *0,0620* | 40,4 ± 18,3 | 45,07 ± 20,32 |  | *0,4686* |
| **LDL-c (mg/dL)** | **> 250** | 85 | 6 | 1,0518 | *0.9247* | 96 | 6 | 0.3438 | *0.0494* | 96 | 6 | 0.4297 | *0.1355* |
|  | **<250** | 149 | 10 |  |  | 55 | 10 |  |  | 55 | 8 |  |  |
| **Family history of dyslipemia** | **yes** | 171 | 12 | 1,000 | *1,0000* | 126 | 12 | 0.5476 | *0.3316* | 126 | 10 | 0.4563 | *0.2156* |
|  | **no** | 57 | 4 |  |  | 23 | 4 |  |  | 23 | 4 |  |  |
| **Family history of CVD/CAD** | **yes** | 148 | 10 | 0.9122 | *0.8635* | 79 | 10 | 1,4768 | *0.4718* | 79 | 4 | 0.3544 | *0.0911* |
|  | **no** | 81 | 6 |  |  | 70 | 6 |  |  | 70 | 10 |  |  |
| **Personal history of CVD/CAD** | **yes** | 54 | 7 | 2,535 | *0.0778* | 22 | 7 | **4,5253** | ***0.0065*** | 22 | 4 | 2,3273 | *0.1835* |
|  | **no** | 176 | 9 |  |  | 128 | 9 |  |  | 128 | 10 |  |  |
