# Supplementary material for: Functional Analysis of 3′UTR Variants at the LDLR and PCSK9 Genes in Patients with Familial Hypercholesterolemia
Source: Hum Mutat. 2024 Feb 8;2024:9964734. doi: 10.1155/2024/9964734 (PMC11918801; doi:10.1155/2024/9964734)
Supplement: Supplementary 3 — Table SPTB3: 3′UTR-LDLR and 3′UTR-PCSK9 variants selected after considering the effect on miRNA binding according to miRanda prediction, allele frequency, and number of carriers. Note: Variants in bold were selected for further functional characterization. [file 9964734.f3.docx]

**Table SPTB3.** *3'UTR-LDLR* and *3'UTR-PCSK9* variants selected after considering the effect on miRNA binding according to miRanda prediction, allele frequency and number of carriers.

|  |  | **Miranda prediction** | | | **GnomaD**  **Allele**  **Frequencies** | **Number**  **of patients** | **Genetic test positive**  **(yes/no)** |
| --- | --- | --- | --- | --- | --- | --- | --- |
|  |  | Added miRNA site | Modified miRNA site | Removed miRNA site |  |  |  |
| **3’UTR-*LDLR* variants** | **c.*19G>A** | 15 | 6 | 1 | 0,000725 | 1 | yes |
|  | c.*223G>A | 2 | 1 | 0 | 0,003 | 1 | yes |
|  | **c.*503C>T** | 11 | 3 | 0 | 0,000319 | 1 | no |
|  | **c.*517C>A** | 11 | 0 | 6 | 0,00229 | 1 | no |
|  | **c.*653G>C** | 21 | 6 | 0 | 0,00172 | 1 | no |
|  | c.*965C>T | 8 | 2 | 0 | 0,00526 | 1 | no |
|  | **c.*1227C>T** | 14 | 2 | 1 | 0,00062 | 1 | yes |
|  | c.*2004C>T | 9 | 4 | 1 | 0,000956 | 1 | yes |
|  | c.*2076dup | 1 | 5 | 0 | 0,00256 | 1 | yes |
|  | c.*2111G>A | 13 | 0 | 0 | 0,00561 | 1 | no |
|  | c.*2132G>A | 8 | 1 | 4 | 0,00207 | 1 | no |
|  | c.*2210T>C | 1 | 4 | 0 | 0,000973 | 2 | yes/no |
|  | c.*2319C>G | 14 | 4 | 1 | 0,0023 | 2 | no |
| **3’UTR-*PCSK9* variants** | **c.*171C>T** | 2 | 2 | 5 | 0,00459 | 4 | 1(yes)/3 (no) |
|  | **c.*234C>T** | 4 | 2 | 8 | 0,00315 | 6 | 3(yes)/3 (no) |
|  | c.*667G>A | 0 | 2 | 9 | - | 1 | yes |
|  | **c.*950C>T** | 4 | 4 | 6 | 0,0000955 | 1 | no |
|  | c.*1052C>T | 0 | 3 | 9 | 0,000574 | 2 | 2 (no) |
|  | c.*1064C>A | 3 | 2 | 11 | 0,00379 | 1 | yes |
|  | c.*1151del | 1 | 7 | 4 | 0,000414 | 1 | yes |
|  | c.*1247_*1250del | 0 | 3 | 2 | 0,000223 | 1 | no |

NOTE: Variants in bold were selected for further functional characterization.
