# Supplementary material for: Functional Analysis of 3′UTR Variants at the LDLR and PCSK9 Genes in Patients with Familial Hypercholesterolemia
Source: Hum Mutat. 2024 Feb 8;2024:9964734. doi: 10.1155/2024/9964734 (PMC11918801; doi:10.1155/2024/9964734)
Supplement: Supplementary 4 — Table SPTB4: validation of the predictions made by the miRanda algorithm on the added miRNA binding sites due to the 3′UTR-LDLR variant c.∗653G > C with three other bioinformatics tools (miRWalk3.0, TargetScan, and miRDB). [file 9964734.f4.docx]

**Table SPTB4**. Validation of the predictions made by the miRanda algorithm on the added miRNA binding sites due to the 3’UTR-*LDLR* variant c.*653G>C with three other bioinformatics tools (mirWalk3.0, TargetScan and miRDB).

| **miRNA add by miRanda prediction** | **Gene** | **3'UTR variants** | **miRWalk v3** | **TargetScan** | **miRDB** | **SUM** |
| --- | --- | --- | --- | --- | --- | --- |
| hsa-miR-181a-2-3p | LDLR | **c.*653G>C** | 0 | 0 | 0 | 0 |
| hsa-miR-185-3p | LDLR | **c.*653G>C** | 1 | 0 | 0 | 1 |
| hsa-miR-1914-5p | LDLR | **c.*653G>C** | 1 | 0 | 0 | 1 |
| hsa-miR-210-3p | LDLR | **c.*653G>C** | 0 | 0 | 0 | 0 |
| hsa-miR-2114-3p | LDLR | **c.*653G>C** | 0 | 0 | 0 | 0 |
| hsa-miR-218-5p | LDLR | **c.*653G>C** | 0 | 0 | 0 | 0 |
| hsa-miR-3164 | LDLR | **c.*653G>C** | 1 | 0 | 0 | 1 |
| hsa-miR-3174 | LDLR | **c.*653G>C** | 1 | 0 | 0 | 1 |
| hsa-miR-34a-5p | LDLR | **c.*653G>C** | 0 | 0 | 0 | 0 |
| hsa-miR-34b-5p | LDLR | **c.*653G>C** | 1 | 0 | 0 | 1 |
| hsa-miR-377-5p | LDLR | **c.*653G>C** | 0 | 0 | 0 | 0 |
| hsa-miR-3929 | LDLR | **c.*653G>C** | 0 | 0 | 0 | 0 |
| hsa-miR-449c-5p | LDLR | **c.*653G>C** | 1 | 0 | 0 | 1 |
| hsa-miR-455-5p | LDLR | **c.*653G>C** | 1 | 0 | 0 | 1 |
| hsa-miR-485-5p | LDLR | **c.*653G>C** | 0 | 0 | 0 | 0 |
| hsa-miR-515-3p | LDLR | **c.*653G>C** | 0 | 0 | 0 | 0 |
| hsa-miR-519e-3p | LDLR | **c.*653G>C** | 1 | 0 | 0 | 1 |
| hsa-miR-544b | LDLR | **c.*653G>C** | 0 | 0 | 0 | 0 |
| hsa-miR-550a-5p | LDLR | **c.*653G>C** | 1 | 0 | 0 | 1 |
| hsa-miR-663b | LDLR | **c.*653G>C** | 0 | 0 | 0 | 0 |
