# Supplementary material for: Functional Analysis of 3′UTR Variants at the LDLR and PCSK9 Genes in Patients with Familial Hypercholesterolemia
Source: Hum Mutat. 2024 Feb 8;2024:9964734. doi: 10.1155/2024/9964734 (PMC11918801; doi:10.1155/2024/9964734)
Supplement: Supplementary 5 — Table SPTB5: validation of the predictions made by the miRanda algorithm on removed miRNA binding sites due to the following 3′UTR-PCSK9 variants: c.∗171C > T, c.∗234C > T, and c.∗950C > T, with three other bioinformatics tools (miRWalk3.0, TargetScan, and miRDB). [file 9964734.f5.docx]

**Table SPTB5**. Validation of the predictions made by the miRanda algorithm on removed miRNA binding sites due to the following 3’UTR-*PCSK9* variants: c.*171C>T, c.*234C>T, and c.*950C>T, with three other bioinformatics tools (mirWalk3.0, TargetScan, and miRDB).

| **miRNA removed by miRanda prediction** | **Gene** | **3'UTR variants** | **miRWalk v3** | **Targetscan** | **miRDB** | **SUM** |
| --- | --- | --- | --- | --- | --- | --- |
| hsa-miR-4269 | PCSK9 | c.*171C>T | 1 | 1 | 0 | 2 |
| hsa-miR-31-5p | PCSK9 | c.*171C>T | 1 | 0 | 0 | 1 |
| hsa-miR-1226-5p | PCSK9 | c.*234C>T | 0 | 1 | 0 | 1 |
| hsa-miR-1294 | PCSK9 | c.*234C>T | 1 | 0 | 0 | 1 |
| hsa-miR-3174 | PCSK9 | c.*234C>T | 0 | 1 | 0 | 1 |
| hsa-miR-4316 | PCSK9 | c.*234C>T | 0 | 0 | 0 | 0 |
