# Supplementary material for: Functional Analysis of 3′UTR Variants at the LDLR and PCSK9 Genes in Patients with Familial Hypercholesterolemia
Source: Hum Mutat. 2024 Feb 8;2024:9964734. doi: 10.1155/2024/9964734 (PMC11918801; doi:10.1155/2024/9964734)
Supplement: Supplementary 6 — Figure SF1: (a) effect of miR-4269 on the expression of the 3′UTR-PCSK9 variant c.∗171C > T and (b) effect of miR-1226 on the expression of the 3′UTR-PCSK9 variant: c.∗234C > T in the luciferase reporter assay. [file 9964734.f6.pdf]

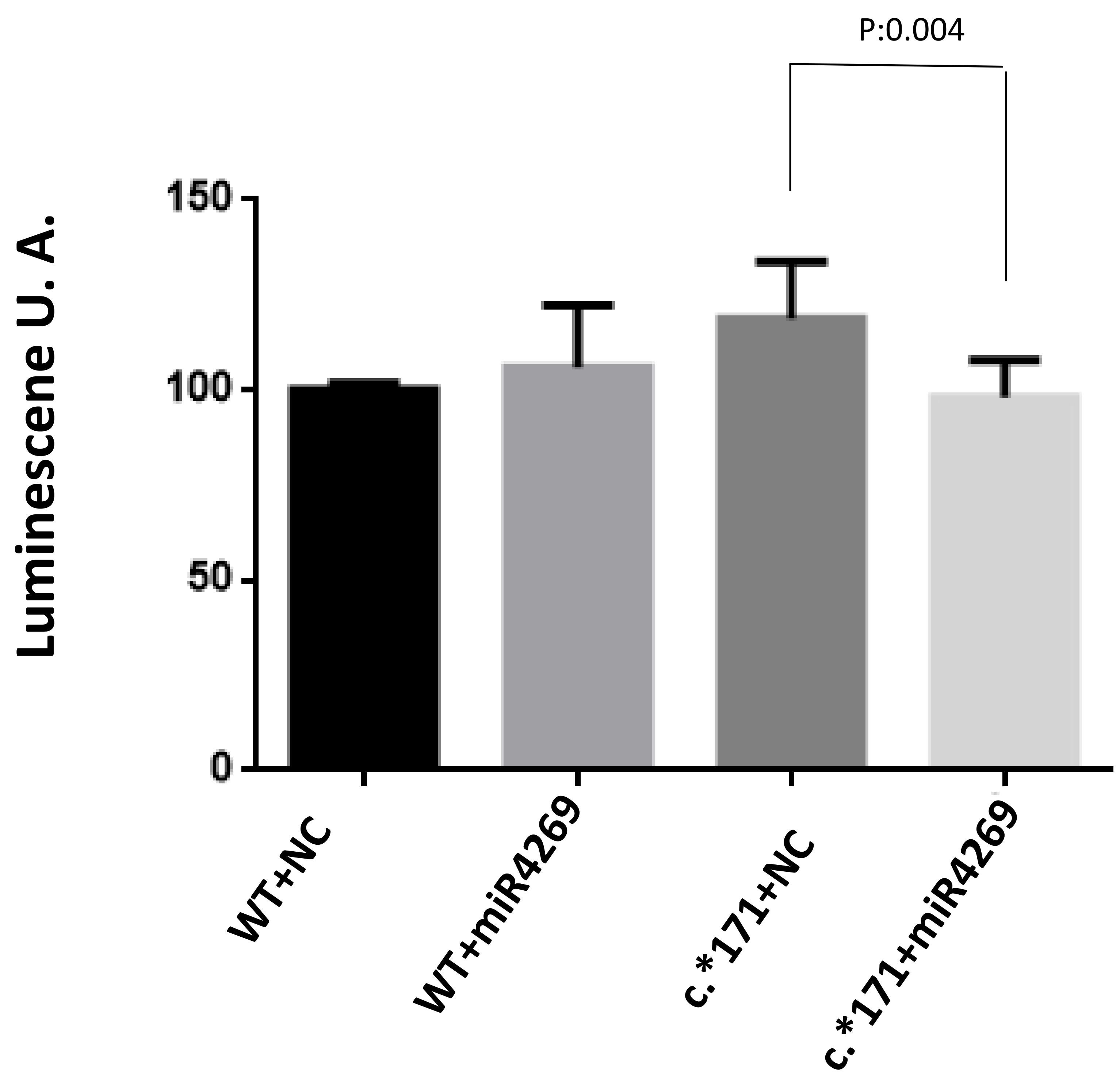

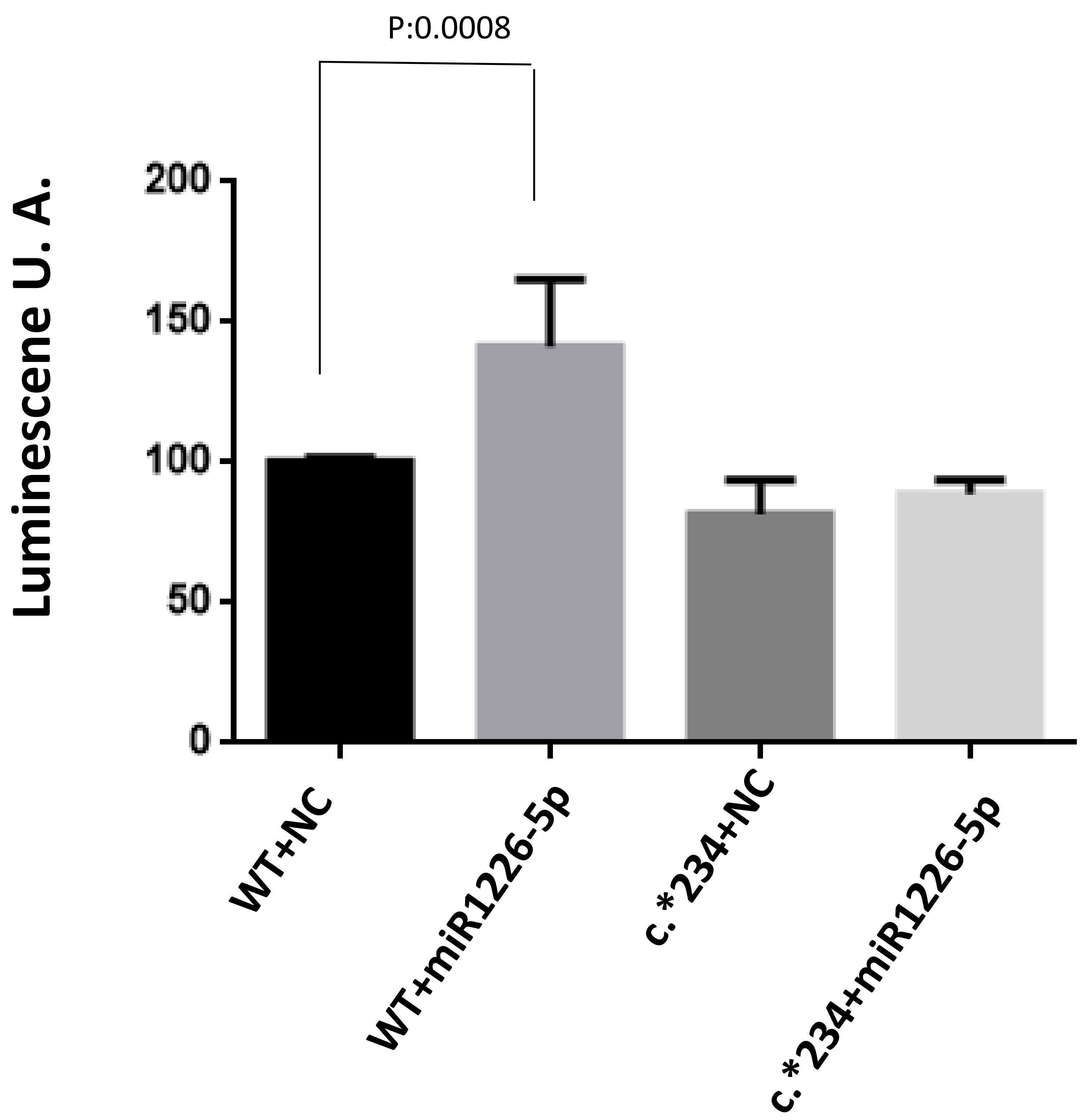

Groups	Luciferase acttivity (%)	Difference between groups	95% confidence interval	P value
PCSK9 3'UTR WT+NC vs<br>PCSK9 3'UTR WT+miR-4269	100.2 ± 0.54<br>105.9 ± 5.47	5.70 ± 5.50	-186.97 to .33	0.3298
c.*171C>T+NC vs<br>c.*171C>T+miR-4269	118.7 ± 4.99<br>98.20 ± 3.23	-20.51 ± 5.95	-33.29 to -7.73	0.004
PCSK9 3'UTR WT+NC vs<br>PCSK9 3'UTR WT+miR-1226-5p	100.2 ± 0.54<br>141,5 ± 7,92	41,25 ± 7,94	22,98 to 59,52	0,0008
c.*234C>T+NC vs<br>c.*234C>T+miR-1226-5p	81,50 ± 4,11<br>88,76 ± 1,72	7,26 ± 4,46	-2,58 to 17,09	0,1324

*WT: wild type; NC negative control; miR-1226: miR1226-5p

Figure SF1. a) Effect of miR-4269 on the expression of the 3'UTR-*PCSK9* variant c.*171C>T and b) effect of miR-1226 on the expression of the 3'UTR-*PCSK9* variant: c.*234C>T in the luciferase reporter assay.
