# Supplementary material for: Functional Analysis of 3′UTR Variants at the LDLR and PCSK9 Genes in Patients with Familial Hypercholesterolemia
Source: Hum Mutat. 2024 Feb 8;2024:9964734. doi: 10.1155/2024/9964734 (PMC11918801; doi:10.1155/2024/9964734)
Supplement: Supplementary 7 — Figure SF2: effect of miR-1226 inhibitor on the expression of the 3′UTR-PCSK9 variant c.∗234C > T versus 3′UTR-PCSK9 WT in the luciferase reporter assay. [file 9964734.f7.pdf]

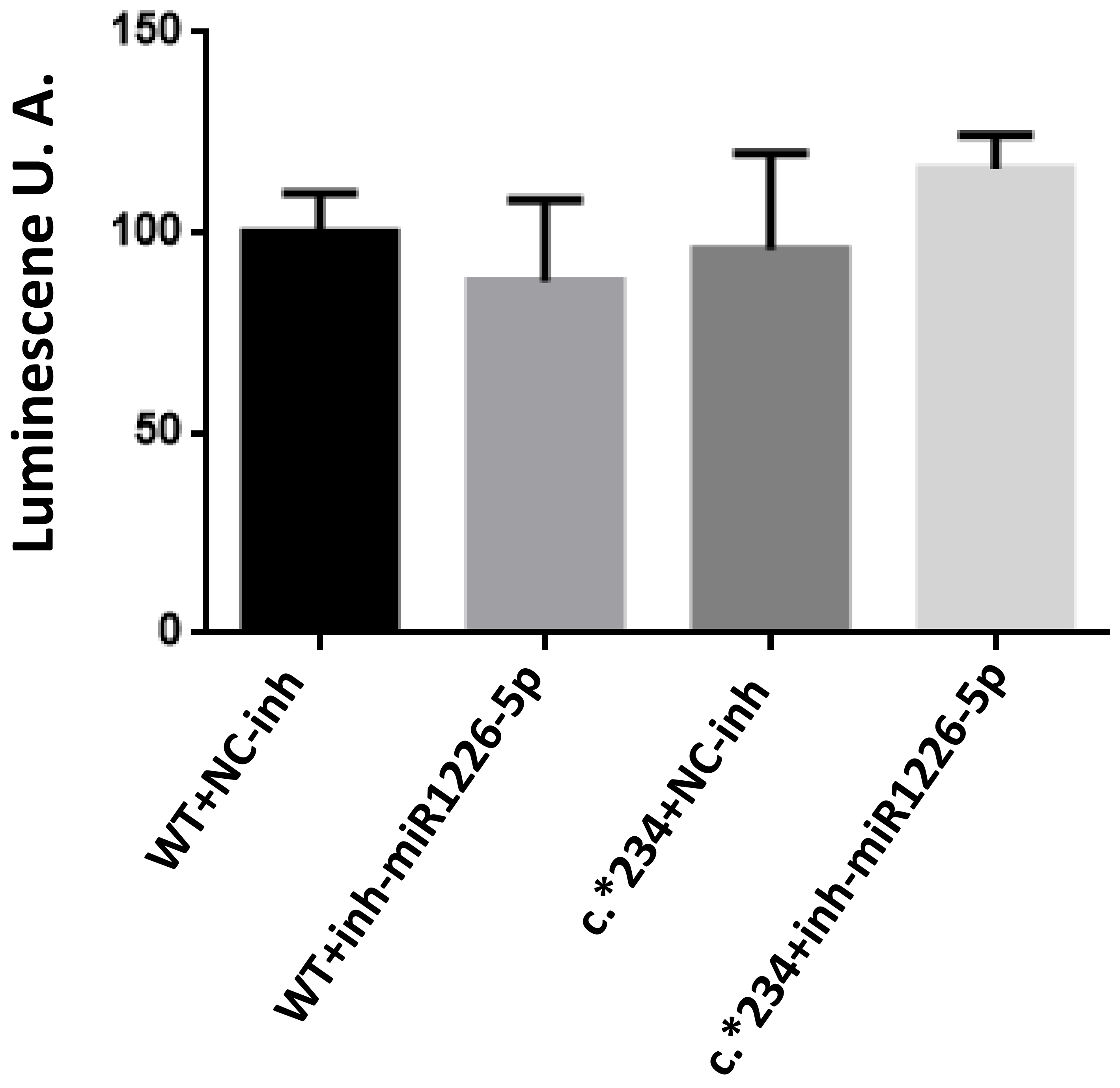

Groups	Luciferase acttivity (%)	Difference between groups	95% confidence interval	P value
PCSK9 3’UTR WT+NC-inh vs<br>3’UTR-PCSK9 WT+inh-miR-1226	99,99 ± 3,14<br>87,76 ± 7,28	-12,23 ± 7,93	-30,00 to 5,54	0,1554
c.*234C>T+NC-inh vs<br>c.*234>T+inh-miR-1226	95,89 ± 8,47<br>116,0 ± 2,66	20,07 ± 8,88	-0,23 to 40,38	0,0521

*WT: wild type; NC-inh negative control inhibitor; inh inhibitor miR-1226: miR1226-5p

Figure SF2. Effect of miR-1226 inhibitor on the expression of the 3’UTR-PCSK9 variant c.*234C>T versus 3’UTR-*PCSK9* WT in the luciferase reporter assay
